# Likely Pathogenic Variants of Ca_v_1.3 and Na_v_1.1 Encoding Genes in Amyotrophic Lateral Sclerosis Could Elucidate the Dysregulated Pain Pathways

**DOI:** 10.3390/biomedicines11030933

**Published:** 2023-03-17

**Authors:** Zsófia Flóra Nagy, Balázs Sonkodi, Margit Pál, Péter Klivényi, Márta Széll

**Affiliations:** 1 Department of Medical Genetics, Albert Szent-Györgyi Medical School, University of Szeged, 6725 Szeged, Hungary; 2 Department of Health Sciences and Sport Medicine, Hungarian University of Sports Science, 1123 Budapest, Hungary; 3 ELKH-SZTE Functional Clinical Genetics Research Group, 6720 Szeged, Hungary; 4 Department of Neurology, Albert Szent-Györgyi Medical School, University of Szeged, 6725 Szeged, Hungary

**Keywords:** amyotrophic lateral sclerosis, pain, Piezo2, Ca_v_1.3, *CACNA1D* gene, Na_v_1.1, *SCN1A* gene, WDR neuron

## Abstract

Amyotrophic lateral sclerosis (ALS) is a lethal multisystem neurodegenerative disease associated with progressive loss of motor neurons, leading to death. Not only is the clinical picture of ALS heterogenous, but also the pain sensation due to different types of pain involvement. ALS used to be considered a painless disease, but research has been emerging and depicting a more complex pain representation in ALS. Pain has been detected even a couple years before the symptomatic stage of ALS, referring to primary pain associated with muscle denervation, although secondary pain due to nociceptive causes is also a part of the clinical picture. A new non-contact dying-back injury mechanism theory of ALS recently postulated that the irreversible intrafusal proprioceptive Piezo2 microinjury could be the primary damage, with underlying genetic and environmental risk factors. Moreover, this Piezo2 primary damage is also proposed to dysregulate the primary pain pathways in the spinal dorsal horn in ALS due to the lost imbalanced subthreshold Ca^2+^ currents, NMDA activation and lost L-type Ca^2+^ currents, leading to the lost activation of wide dynamic range neurons. Our investigation is the first to show that the likely pathogenic variants of the Ca_v_1.3 encoding *CACNA1D* gene may play a role in ALS pathology and the associated dysregulation or loss of the pain sensation. Furthermore, our reanalysis also shows that the *SCN1A* gene might also contribute to the dysregulated pain sensation in ALS. Finally, the absence of pathogenic variants of Piezo2 points toward the new non-contact dying-back injury mechanism theory of ALS. However, molecular and genetic investigations are needed to identify the functionally diverse features of this proposed novel critical pathway.

## 1. Introduction

Amyotrophic lateral sclerosis (ALS) is an adult-onset lethal multisystem neurodegenerative disease associated with progressive loss of motoneurons and death, mostly within 3–5 years [1]. The pathogenesis of ALS is substantially unknown despite it being first noticed more than 150 years ago by a great French neurologist, Jean-Martin Charcot [2]. The majority of ALS cases are sporadic, with around 90% prevalence [3], and only about 10% of cases represent familial ones [4,5]. The genetic background of ALS is rather diverse, because heritability is estimated to be 50% even among sporadic patients [6] with non-coding DNA involvement [7,8,9]. It is important to note that non-coding does not mean that they are not carrying important signals vital in order to maintain homeostasis; however, when this non-coding DNA signaling is dysregulated, then it could have a contributing role in the pathogenesis of ALS. Moreover, these non-coding DNA functions are rather cell-type-specific [9,10]; therefore, identifying their function and disfunction could enhance our understanding of neuroscience. In essence, there is still a great need for further revelation concerning the genetics of ALS, and for this reason, a significant amount of further genetic research has to be done for advancement. Currently, more than 130 genes have been associated with ALS according to the ALS Online Database (http://www.alsod.ac.uk, accessed on 16 December 2022). However, only a fraction of the genes linked to ALS have a causal relationship with the disease. Most of the genes are either susceptibility factors or genes associated with other neurodegenerative conditions which have symptoms that might mimic ALS.

Presymptomatic somatosensory involvement with sensory circuit dysfunction is shown in the ALS disease process [11,12,13]. Not only is the clinical picture of ALS heterogenous, but also the pain sensation due to different types of pain involvement [14]. ALS used to be considered not only a disease predominantly of the motor neurons, but also a painless disease; however, research has been emerging in the past 10 years depicting a more complex pain representation in ALS [15]. Pain has been detected even 1–2 years before the symptomatic stage of ALS [14], as the primary pain associated with muscle denervation [16] and the secondary pain due to nociceptive causes are also represented in ALS [17].

A new non-contact dying-back injury mechanism theory of ALS recently postulated that irreversible intrafusal proprioceptive Piezo2 microinjury could be the primary damage [18,19] and that it is suggested to be a principal transcription activator [19], therefore letting the underlying genetic variants and cell-type-specific non-coding DNA variants become more apparent. Moreover, this Piezo2 primary damage is also proposed to dysregulate the primary pain pathways in the spinal dorsal horn of ALS due to lost imbalanced subthreshold Ca^2+^ currents and N-methyl-d-aspartate (NMDA) activation [19,20]. Indeed, animal research shows that the loss-of-function mutations in Piezo2 lead to loss of the pain sensation [21]. Hence, the irreversible Piezo2 microinjury-induced lost imbalanced subthreshold leakage Ca^2+^ currents, lost NMDA activation and resultant lost L-type Ca^2+^ currents could lead to lost activation of wide dynamic range (WDR) neurons, based on the findings of Aguiar et al. [19,22]. Activated WDR neurons have been long suspected to be the gatekeepers according to the gate control theory of pain [23,24]. A current opinion piece postulated that the loss of these signaling pathways, leading to miswired proprioception, could be one reason why the primary pain is so heterogenous in ALS and why it used to be considered a painless disease, not to mention that the irreversible Piezo2 microinjury could constantly activate transcription pathways [19].

Correspondingly, the goal of our study was to reanalyze the potential pathogenic gene variants from our previous ALS study pertaining to the aforementioned pain signaling pathways, with a special focus on the L-type voltage-gated calcium channel, namely the Ca_v_1.3 ion channel, encoded by the voltage-gated channel subunit alpha1 D (*CACNA1D*) gene. *CACNA1D* gene mutations are known to cause aldosteronism and neuromuscular abnormalities, not to mention neurological abnormalities in autism spectrum disorder and epilepsy, and in primary aldosteronism, seizures and neurological abnormalities (PASNA) [25,26], although no relation to ALS has been reported yet. Indeed, it is indicative of our theory that a recent study presented in autism—which also used to be considered a painless disorder [27]—exhibits sensory hypersensitivity to daily stimuli and to experimental pain [28]. Furthermore, we also aimed to reanalyze the pathomechanistic pathways downstream, including the genes of the Piezo2 channel as the site of the primary damage, as explained through the new non-contact dying-back injury mechanism theory of ALS [18,20].

## 2. Results

### 2.1. Analysis of the Ca_v_1.3 and Ca_v_1.2 Channels Encoding Gene

By reanalyzing the whole exome sequencing data of 21 ALS patients, a total of 6 *CACNA1D* variants were revealed: one pathogenic variant confirmed by functional studies, one likely pathogenic, three variants of uncertain significance and a further likely benign variant. All six variants were present in separate patients respectively; no patients were identified to carry more than one *CACNA1D* variant. Table 1 summarizes the variants identified in our study.

**Figure 1:**
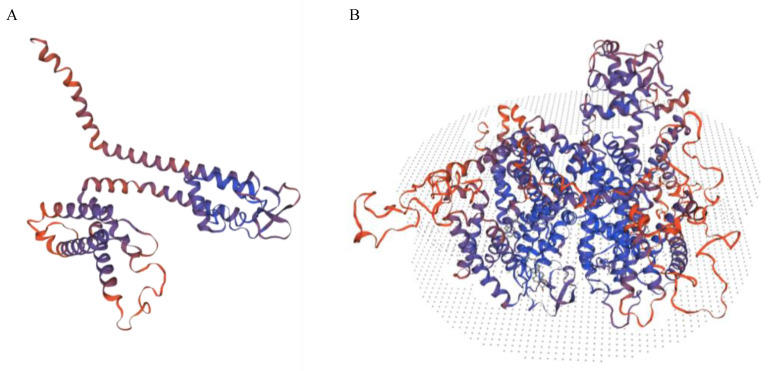
This figure shows the predicted structure of p.Arg510Ter (**A**) compared to the structure of the wild type (**B**) based on homology modelling by Swiss Model (https://swissmodel.expasy.org, accessed on 15 February 2023) [30].

The p.Arg510Ter variant is missing from the largest population genetics databases. The variant leads to a premature stop codon and may result in the loss of more than 3/4 of the wild-type protein (see Figure 1). The variant affects the II repeat sequence. According to the American College of Medical Genetics and Genomics (ACMG) guidelines, the variant may be reported as likely pathogenic. The variant was previously reported in aldosterone-producing cell clusters, the precursor to an aldosterone-producing adenomas, and the somatic mutations accumulate in an aging-dependent manner [29].

The p.Phe747Leu variant fulfills the following ACMG criteria: PM2 moderate, PM1 pathogenic supporting, the PP2 pathogenic supporting and PP3 pathogenic supporting. The variant is also absent from the most relevant population genetic databases, and it is predicted to be deleterious by multiple in silico analysis tools. The role of the variant was investigated in aldosterone-producing adenomas as well as in hyperaldosteronism, and it was found to be disease-causing by a gain-of-function mechanism [31].

A pathogenic-leaning variant of uncertain significance, p.Glu162Val, of the *CACNA1D* gene was identified in our study. The variant lays on the translational start of exon 4 and is predicted to alter splicing by the dbscSNV RF database. Splicing defects could disturb the connection of the domains of the amino acid 162 borders and the extracellular and transmembrane domains of the repeat I sequence.

The p.Arg1979Gln variant of the calcium *CACNA1D* gene has been submitted to the ClinVar database as a variant of uncertain significance, and it has an allele frequency of 0.0053% among non-Finnish Europeans. The variant is located in the cytoplasmic part of the protein.

An additional variant of unknown significance was also identified in the cytoplasmic-located region of the protein, p.Gly1841Arg. The variant has not been included in the major population genetic databases.

It is important to note that no variants of interest were uncovered during the analysis of the *CACNA1C* gene.

### 2.2. Analysis of the Piezo, Na_v_ Channel, NMDA, GABA and Glycine Receptor Encoding Genes

The reanalysis of the whole exome sequencing data of the same 21 selected ALS patients [32] covered the following genes: *PIEZO1*, *PIEZO2*, *SCN1A*, *SCN8A*, and *SCN9A*. It is noteworthy that the *SCN1A*, *SCN8A*, and *SCN9A* genes encode the Na_v_1.1, Na_v_1.6 and Na_v_1.7 channels, respectively, which could be found on proprioceptors. The contribution of the Peizo2 and Na_v_1.1 channels in the static encoding of proprioception has been demonstrated [33,34]. Moreover, it has been theorized that the combination of Piezo2, Na_v_1.6, Na_v_1.7 and/or other channels, such as the glutamate receptors, ASIC and ENaC ones, are involved in the dynamic encoding of proprioception [33]. In summation, throughout the 21 samples, we identified 46 different variants (22 in *PIEZO1*, 9 in *PIEZO2*, 7 in *SCN1A*, 5 in *SCN9A* and 5 in *SCN8A*).

Out of the identified 46 variants, we detected 4 relevant variants in the *SCN1A* gene. Table 2 shows the variants of interest identified in the *SCN1A* gene. Variants of the gene have been associated with a severe form of epileptic encephalopathy, namely Dravet syndrome, and with generalized epilepsy with febrile seizures in the scientific literature [35,36]. Accordingly, a known reported pathogenic variant was identified, p.S228P. The variant is absent from population genetic databases, and it is predicted to be pathogenic by the most relevant predictive in silico tools. Furthermore, the variant fulfills the PP5, PP3, PM2, PM5, PM1and PP2 criteria of pathogenicity [37]. The missense variant is located in the S4 helical transmembrane unit of the repeat I region. The variant is located in a very conserved region of the protein and has a phyloP score of 9.322, which also predicts the deleterious effect of the variant. The region might be considered a mutational hotspot since variants affecting the positions three nucleotides upstream and two and five nucleotides downstream, respectively, from the position of the above-mentioned variant have also been found pathogenic.

A formerly reported likely pathogenic variant, the p.T398M variant, was also detected in one patient. It has been submitted to ClinVar as likely pathogenic even though the variant fulfills the criteria for a pathogenic variant as well. The missense variant was very rarely identified in people of European origin. The p.T398M variant resides in the first repeat domain and lies very close to a pore-forming intramembrane sequence. The variant was previously described in a female with focal drug-resistant epilepsy possibly originating from hippocampal sclerosis [38].

The p.R1927G variant is a missense variant, and it has not been identified in the most relevant population genetic databases either. According to the ACMG classification guidelines, the variant is likely pathogenic [37]. Moreover, prediction tools such as Revel, Varity and MetaLR suppose its pathogenicity. The variant affects the cytoplasmically located IQ domain of the protein that is responsible for calmodulin binding [39].

The p.L863F variant may also be classified as likely pathogenic. The variant is also missing from wide-ranging population genetic databases. The variant is located just on the translational start of exon 17/29, and a cytosine to adenine change is likely to result in a splice donor loss according to SpliceAI. The amino acid position 863 is involved in the formation of the transmembrane sequence of the repeat domain II, a key component of the protein. 

It is important to note that no variants of interest were uncovered during the analysis of the *PIEZO1*, *PIEZO2*, *SCN8A*, *SCN9A*, NMDA receptor encoding *GRIN2A*, gamma-aminobutyric acid (GABA) receptor encoding *GABRA1* and glycine receptor encoding *GLRA1* genes.

## 3. Discussion

**Figure 2:**
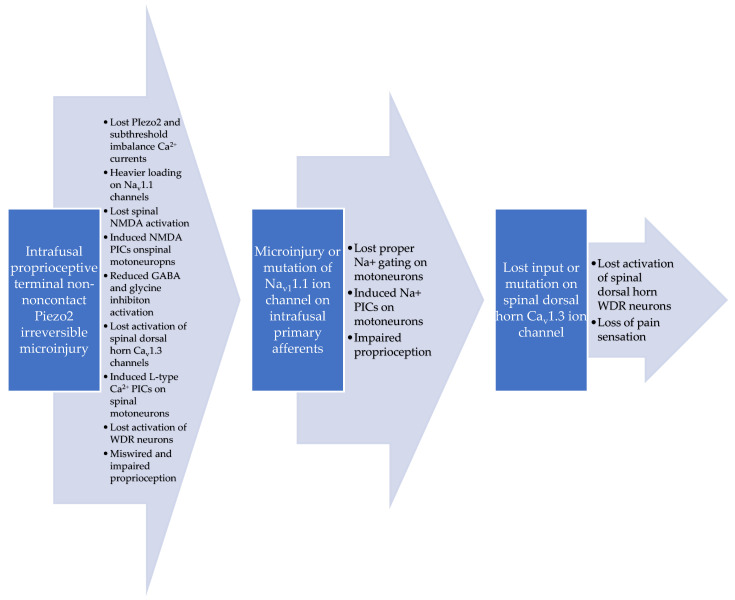
Proposed dysregulated pain pathways in ALS based on intrafusal noncontact proprioceptive terminal Piezo2 irreversible microinjury-induced miswired proprioception [19].

*CACNA1D* gene variants have been recently identified in another neurodegenerative disease, namely in Parkinsonism. It is hypothesized that gain-of-function variants of the Ca^2+^ channels make the neurons more susceptible to cellular stress and that the overload of Ca^2+^ signaling may even lead to cell death [40]. Ca_v_1.3 voltage-gated L-type Ca^2+^ channels can be found postsynaptically in neurons contributing to neuronal firing and plasticity, although they also play a role in cardiac pacemaking [41]. In addition, it is theorized that the dysfunctional spinal microcircuits of ALS are progressively interfering with the synchronization of central pattern generators (CPG) [18], such as locomotion, and the aforementioned rhythmic firing feature of the Ca_v_1.3 ion channels could be involved in this process. It is noteworthy that animal research demonstrates that Piezo2 indeed has a role in synchronizing neural networks supraspinally [42], and it might be the case spinally as well. Hence, the lost function of excitatory Piezo2 in ALS could lead to the theorized impairment of the spinal synchronization of CPGs and to the lost function of the spinal Ca_v_1.3 ion channels. It is indicative of this lost spinal Ca_v_1.3 ion channel function that a recent study with a mouse model for ALS observed enhanced persistent inward currents (PICs) on the spinal motoneurons, including L-type Ca^2+^ currents [43]. Inhibition of the overactivated calcium channels seems to be a promising drug target for neurodegenerative diseases with the underlying functional defect [44]. However, trials are still in the early phases [45]. Moreover, it is important to note that Riluzole, a longtime medication for only ALS, has a neuroprotective effect and multiple drug action. Among these, it could transiently rectify the Ca^2+^ currents and NMDA receptors [46], not to mention that it also inhibits the persistent Na^+^ currents [47]. It is notable that it is theorized that part of the miswired proprioceptive input in ALS is lost NMDA receptor activation spinally, with resultant increased NMDA PICs on the motoneurons, in addition to increased Na^+^ PICs due to the noncontact proprioceptive terminal Piezo2 irreversible microinjury [19,48] (see Figure 2). Nevertheless, these beneficial effects of Riluzole are minimal [49] and not sustainable in ALS due to its rapid progression in the pathomechanism.

It is interesting to note that autonomic disbalance could elevate the risk of abnormal cardiac rhythm or even sudden cardiac death in ALS [50,51]. Dysautonomia is suggested to be the direct result of the proprioceptive Piezo2 irreversible microinjury in ALS [18,19], not to mention the finding that in fact the loss of Piezo2 vagal neurons put an end to baroreceptor sensing and reflex [52]. Even more importantly, increased sympathetic loading triggered automaticity, which is suggested to be not analogous with baroreflex-induced cardiac autonomic outflows measured by heart rate variability [53], could involve the Ca_v_1.3 ion channels in the sinoatrial pacemaker cells based on animal research [54]. Indeed, early sympathetic overactivity is demonstrated in ALS, and the cause is attributed to disruption of the baroreflex pathways [55]. Therefore, intact Ca_v_1.3 ion channels could also have special cardiac relevance under the sympathetic overactivity of ALS.

Our novel finding, that likely pathogenic variants of the Ca_v_1.3 encoding *CACNA1D* gene may play a role in the ALS pathomechanism, could have special relevance in the dysregulated or lost pain pathways in the spinal dorsal horn. Pain has been reported up to two years before the onset of ALS symptoms [14]. Primary pain is also present once the symptoms become obvious in the ALS disease process, and this primary pain is considered to be the consequence of muscle denervation [16]. Moreover, secondary pain due to nociceptive causes is also part of the clinical picture of ALS [17]. Hence, the pain sensation shows a diverse clinical picture in ALS without knowing the exact mechanism of it. The dysregulated pain sensation in ALS is noteworthy, especially in light of the fact that it used to be considered a painless disease.

The recent ALS non-contact dying-back injury mechanism theory postulated that aging-associated non-contact microinjury of the intrafusal proprioceptive terminal Piezo2 channel could be irreversible due to the complete loss of Piezo2 mechano- and force-gating [20]. Moreover, the concomitant impairment of the glutamate vesicular release could result in vesicular glutamate transporter 1 (VGLUT1) synaptic disconnection and NMDA PIC inducement on the motoneurons, and in spinal NMDA receptor activation due to this microinjury [56]. However, the irreversible fashion of this microdamage in ALS also means that NMDA receptor activation is lost over time. It is noteworthy that increased NMDA receptor activation has been proposed recently as the gatekeeper in accordance with the gate control theory of pain [18]; therefore, the lost NMDA receptor activation could also explain the dysregulated pain sensation. In addition, this delay may explain the presymptomatic pain in ALS and the dysregulated pain representation later in the disease process. Nevertheless, the subthreshold imbalanced leakage Ca^2+^ currents are suggested to also be lost due to the complete loss of mechano- and force-gating at the intrafusal proprioceptive terminal Piezo2, and that could lead to lost activation of the L-type Ca_v_1.3 calcium channels in the spinal dorsal horn [19]. It has been demonstrated that the activation of NMDA receptors in conjunction with L-type calcium currents and nonspecific cationic currents is indeed needed for WDR neuron activation in the dorsal horn [22]. Since the activation of the WDR neurons in the spinal dorsal horn has been long insinuated in reference to the gate control theory of pain [23,24], as have activated NMDA receptors too [18], the loss of these activation mechanisms could explain the dysregulated and lost primary pain sensation in ALS. It is important to note again that the pain sensation and sensitization are indeed lost due to loss-of-function mutations in Piezo2 [21]. An in-depth understanding of the potential role of Piezo2 in pain sensation could be achieved from a review article in reference to delayed-onset muscle soreness [48], because this is the mechanism that is suggested to be lost in ALS. Moreover, it is also indicative that Piezo modulates the L-type calcium currents [57]. Furthermore, it is known that the Ca_v_1.2 and Ca_v_1.3 calcium channels play a role in the activation of the dorsal horn WDR neurons. Ca_v_1.3 is the one instigating the wind-up in coordination with the activated NMDA receptors [58], not to mention that the role of the WDR neurons is noted in pain sensitization in the spinal dorsal horn [59].

In addition, an aforementioned new study presented that not only were the PICs enhanced on the spinal motoneurons in the ALS mouse model, but it was also accompanied by reduced GABA inhibition on the motoneurons [43]. It is notable that GABAergic and/or glycinergic presynaptic modulation of the spinal proprioceptive input, in addition to postsynaptic modulation by interneurons, have a role in the control of spinal reflexes [60,61,62]. It is interesting to note that reduced input of GABAergic interneurons is associated with reduced input on WDR neurons [63]. Therefore, not only the lost activation of the L-type Ca_v_1.3 calcium channels, in association with the lost Piezo2 functionality and lost NDMA activation, could impede the activation of the spinal WDR neurons, but as a result, the reduced GABAergic inhibition cannot activate the WDR neurons either (see Figure 2). This reduced spinal postsynaptic GABAergic inhibition on the motoneurons could be the direct result of the loss due to the intrafusal primary afferent input at the spinal and supraspinal levels, as could be the case in glycinergic inhibition as well. Indeed, decreased GABAergic inhibition is present supraspinally in ALS [64] and possibly at the spinal level; therefore, GABA cannot buffer the glutamate excitotoxicity-induced motoneuron damage. Likewise, insufficient glycinergic inhibition spinally is present early in the disease pathomechanism of ALS [65]. However, these spinal reduced synaptic inhibitory inputs could develop over time in the early phase of ALS due to the depleted compensatory mechanisms induced by the presymptomatic inhibitory dysfunction, as was modelled by computer stimulations [66]. Correspondingly, it is not surprising that no variants of significance were detected during the analysis of the GABA receptor encoding *GABRA1* and glycine receptor encoding *GLRA1* genes. The current authors suggest that the lost proper input from the irreversibly microinjured primary afferents decreases the GABAergic and glycinergic inhibition. Nevertheless, these GABAergic and glycinergic inhibitory pathways cannot activate the WDR neurons, although on the other hand, they could become depleted in a compensatory fashion due to the irreversible Piezo2 microinjury at the proprioceptive primary afferent terminals and the resultant miswired proprioception (see Figure 2).

In summary, our findings on the likely loss-of-function pathogenic variants [67] of the Ca_v_1.3 encoding *CACNA1D* gene in ALS could be indicative of the irreversible Piezo2 microdamage-derived dysregulated or lost pain pathways in the spinal dorsal horn of ALS, although further functional analyses are needed to prove the link between the detected variants and the altered cellular functions as well.

The role of the Na_v_1.1 channel encoding genes with their several mutations is known in brain disorders, such as in epilepsy, migraine or autism as well [68,69]. Voltage-gated sodium channels contain four homologous domains, each of which contains six transmembrane regions [70]. Both of the pathogenic variants identified in our study are located in a transmembrane segment of the protein; thus, they likely disrupt the physiological conduction of the ion channel. Loss-of-function variants of the gene are known to result in a genetic epilepsy syndrome phenotype, while gain-of-function variants of the gene are associated with hemiparetic aural migraines [71]. FunNCion (https://funnc.shinyapps.io/shinyappweb/, accessed on 15 February 2023) is an in silico tool to assess the probability of a variant being a loss- or gain-of-function variant in a voltage-dependent sodium/calcium channel gene by a Receiver Operating Characteristic curve. Two of the identified variants are predicted to be pathogenic (p.S228P, p.L863F) with a probability of more than 0.89, while all four variants are thought to be loss-of-function variants. 

Nevertheless, Na_v_1.1 channels are also present in the periphery in intrafusal proprioceptive terminals, not to mention their role when they take over the firing propagation of inactivated Piezo2 channels during prolonged muscle stretching [33]. This pathway is important in reference to our investigation since it is involved in the new non-contact dying-back injury mechanism theory of ALS [18,19,20]. Last but not least, these Na_v_1.1 channels have an indispensable role in mechanical pain sensation [33]. Accordingly, the current authors postulate that the irreversible Piezo2 microinjury disrupts the static phase firing sensory encoding propagation by the Na_v_1.1 channels; hence, the mechanical pain detection could be dysregulated in ALS. It is noteworthy again that the loss-of-function mutation in Piezo2 indeed result in loss of pain and sensitization [21]. Indicative of the disruption of static phase sensory encoding propagation by the Na_v_1.1 channels is the increased magnitude of the Na^+^ PICs in an ALS mouse model [43] (see Figure 2).

In summary, all the above-mentioned Na_v_1.1 variants could result in damage to the ion channel and disrupt its physiological function, not to mention their potential role in pain dysregulation in ALS. However, further functional studies will be needed to confirm their supported pathogenicity.

## 4. Materials and Methods

A total of 21 non-related patients of Hungarian origin who were diagnosed with ALS were recruited for our study. All the patients fulfilled the revised El-Escorial and Awaji scheme criteria for ALS and gave their informed consent to participate in the study [72,73]. No patients reported a positive family history of ALS or any other neurodegenerative condition. Table 3 contains demographic data concerning the enrolled patients.

Whole exome sequencing was carried out, which we have described in detail in one of our previous reports [32]. The whole exome sequencing was performed on an Illumina NextSeq 500 device, and an average per base coverage of 71x was reached. A Genome Analysis Toolkit (GATK) was implemented for variant calling purposes, and the ANNOVAR software (version 17 July 2017) tool was used for the variant annotation.

By reanalyzing the whole exome sequencing data, we were able to evaluate the variants of the *CACNA1D* and the *PIEZO1*, *PIEZO2*, *SCN1A*, *SCN8A*, *SCN9A*, *GRIN2A*, *GABRA1*, *GABRA1* and *GLRA1* genes. The variants were classified according to the 2015 guideline of the ACMG [37]. The detected variants were assessed with the help of comprehensive population genetic databases (gnomAD, 1000 Genomes) and predictive tools (SpliceAI, PolyPhen2, SIFT, MutationTaster, REVEL) to reveal their possible significance. Previous scientific publications were also guiding our assessment. 

## 5. Conclusions

We are the first to report that the likely pathogenic variants of the Ca_v_1.3 encoding *CACNA1D* gene may play a role in ALS pathogenesis and be associated with dysregulated pain sensation or loss of pain sensation. This finding is indicative of a novel pathomechanistic pathway explained through the new non-contact dying-back injury mechanism theory of ALS, namely the irreversible Piezo2 microinjury is the primary damage that could reveal the underlying genetic and environmental risk factors in ALS. We also identified *SCN1A* gene variants that could contribute to the dysregulated pain sensation in ALS. Another important finding was that we excluded the existence of pathogenic variants of Piezo channel encoding genes in our sample, pointing in the direction of the new non-contact dying-back injury mechanism theory of ALS where the primary damage is intrafusal proprioceptive terminal Piezo2 microinjury based. Further molecular and genetic investigations are needed in order to identify the functionally diverse features of this proposed novel critical pathway.

## Figures and Tables

**Table 1:** Identified *CACNA1D* variants of 21 ALS patients.

Gene	Transcription Number	Variant	MAF in Non-Finnish European Population in Genome Aggregation Database	American College of Medical Genetics and Genomics (ACMG) Classification	Sample Number
*CACNA1D*	NM_001128839	c.G5864A, p.Arg1955Gln	0.0053%	Variant of unknown significance (VUS)	42r
*CACNA1D*	NM_001128839	c.A485T, p.Glu162Val	0	Variant of unknown significance (VUS)	48r
*CACNA1D*	NM_001128839	c.C1528T, p.Arg510Ter	0	Likely pathogenic	74r
*CACNA1D*	NM_001128839	c.C2241A, p.Phe747Leu	0	Variant of unknown significance (VUS)	64r
*CACNA1D*	NM_001128839	c.G5449A, p.Gly1817Arg	0	Variant of unknown significance (VUS)	54r
*CACNA1D*	NM_001128839	c.5694_5696del, p.Phe1899delfsTer239	0.4556%	Benign	62r

**Table 2:** Identified *SCN1A* variants of 21 ALS patients.

Gene	Transcription Number	Variant	MAF in Non-Finnish European Population in Genome Aggregation Database	American College of Medical Genetics and Genomics (ACMG) Classification	Sample Number
*SCN1A*	NM_001165963	c.A5779G, p.R1927G	0	Likely pathogenic	79r
*SCN1A*	NM_001165963	c.G2589T, p.L863F	0	Pathogenic	71r
*SCN1A*	NM_001165963	c.C1193T, p.T398M	0.0016%	Likely pathogenic	46r
*SCN1A*	NM_001165963	c.T682C, p.S228P	0	Pathogenic	55r

**Table 3:** Baseline characteristics of the 21 Hungarian patients involved in the study.

Minimum age	40
Maximum age	73
Average age	60.0526
Standard deviation	8.8095
Number of females	10
Number of males	11

## Data Availability

The data presented in this study are available on request from the corresponding author.

## Abbreviations

ACMG	American College of Medical Genetics and Genomics
ALS	Amyotrophic lateral sclerosis
CACNA1D	Voltage-gated channel subunit alpha1 D
CPG	Central pattern generators
GABA	Gamma-aminobutyric acid
NMDA	N-methyl-d-aspartate
PASNA	Primary aldosteronism, seizures and neurological abnormalities
PIC	Persistent inward currents
VGLUT1	Vesicular glutamate transporter 1
WDR	Wide dynamic range

## References

[1] Pasinelli P., Brown R.H. (2006). Molecular biology of amyotrophic lateral sclerosis: Insights from genetics. Nat. Rev. Neurosci..

[2] Charcot J.-M.J.A. (1869). Deux Cas d’atrophie Musculaire Progressive: Avec lÈsions de la Substance Grise et des Faisceaux antÈrolatÈraux de la Moelle ÈpiniËre.

[3] Chen S., Sayana P., Zhang X., Le W. (2013). Genetics of amyotrophic lateral sclerosis: An update. Mol. Neurodegener..

[4] Kurland L.T., Mulder D.W. (1955). Epidemiologic investigations of amyotrophic lateral sclerosis. 2. Familial aggregations indicative of dominant inheritance. I. Neurology.

[5] Kurland L.T., Mulder D.W. (1955). Epidemiologic investigations of amyotrophic lateral sclerosis. 2. Familial aggregations indicative of dominant inheritance. II. Neurology.

[6] Ryan M., Heverin M., McLaughlin R.L., Hardiman O. (2019). Lifetime Risk and Heritability of Amyotrophic Lateral Sclerosis. JAMA Neurol..

[7] Van Rheenen W., Shatunov A., Dekker A.M., McLaughlin R.L., Diekstra F.P., Pulit S.L., van der Spek R.A., Vosa U., de Jong S., Robinson M.R. (2016). Genome-wide association analyses identify new risk variants and the genetic architecture of amyotrophic lateral sclerosis. Nat. Genet..

[8] Nicolas A., Kenna K.P., Renton A.E., Ticozzi N., Faghri F., Chia R., Dominov J.A., Kenna B.J., Nalls M.A., Keagle P. (2018). Genome-wide Analyses Identify KIF5A as a Novel ALS Gene. Neuron.

[9] Zhang S., Cooper-Knock J., Weimer A.K., Shi M., Moll T., Marshall J.N.G., Harvey C., Nezhad H.G., Franklin J., Souza C.D.S. (2022). Genome-wide identification of the genetic basis of amyotrophic lateral sclerosis. Neuron.

[10] Heinz S., Romanoski C.E., Benner C., Glass C.K. (2015). The selection and function of cell type-specific enhancers. Nat. Rev. Mol. Cell Biol..

[11] Vaughan S.K., Kemp Z., Hatzipetros T., Vieira F., Valdez G. (2015). Degeneration of proprioceptive sensory nerve endings in mice harboring amyotrophic lateral sclerosis-causing mutations. J. Comp. Neurol..

[12] Held A., Major P., Sahin A., Reenan R.A., Lipscombe D., Wharton K.A. (2019). Circuit Dysfunction in SOD1-ALS Model First Detected in Sensory Feedback Prior to Motor Neuron Degeneration Is Alleviated by BMP Signaling. J. Neurosci..

[13] Brownstone R.M., Lancelin C. (2018). Escape from homeostasis: Spinal microcircuits and progression of amyotrophic lateral sclerosis. J. Neurophysiol..

[14] Chio A., Mora G., Lauria G. (2017). Pain in amyotrophic lateral sclerosis. Lancet Neurol..

[15] Kwak S. (2022). Pain in amyotrophic lateral sclerosis: A narrative review. J. Yeungnam Med. Sci..

[16] Miller T.M., Layzer R.B. (2005). Muscle cramps. Muscle Nerve.

[17] Smart K.M., Blake C., Staines A., Doody C. (2010). Clinical indicators of ‘nociceptive’, ‘peripheral neuropathic’ and ‘central’ mechanisms of musculoskeletal pain. A Delphi survey of expert clinicians. Man. Ther..

[18] Sonkodi B. (2021). Delayed Onset Muscle Soreness (DOMS): The Repeated Bout Effect and Chemotherapy-Induced Axonopathy May Help Explain the Dying-Back Mechanism in Amyotrophic Lateral Sclerosis and Other Neurodegenerative Diseases. Brain Sci..

[19] Sonkodi B. (2023). Miswired Proprioception in Amyotrophic Lateral Sclerosis in Relation to Pain Sensation (and in Delayed Onset Muscle Soreness)&mdash;Is Piezo2 Channelopathy a Principal Transcription Activator in Proprioceptive Terminals Besides Being the Potential Primary Damage?. Life.

[20] Sonkodi B., Hortobágyi T. (2022). Amyotrophic lateral sclerosis and delayed onset muscle soreness in light of the impaired blink and stretch reflexes – watch out for Piezo2. Open Med..

[21] Szczot M., Liljencrantz J., Ghitani N., Barik A., Lam R., Thompson J.H., Bharucha-Goebel D., Saade D., Necaise A., Donkervoort S. (2018). PIEZO2 mediates injury-induced tactile pain in mice and humans. Sci. Transl. Med..

[22] Aguiar P., Sousa M., Lima D. (2010). NMDA channels together with L-type calcium currents and calcium-activated nonspecific cationic currents are sufficient to generate windup in WDR neurons. J. Neurophysiol..

[23] Chung J.M., Surmeier D.J., Lee K.H., Sorkin L.S., Honda C.N., Tsong Y., Willis W.D. (1986). Classification of primate spinothalamic and somatosensory thalamic neurons based on cluster analysis. J. Neurophysiol..

[24] Price D.D., Dubner R. (1977). Mechanisms of first and second pain in the peripheral and central nervous systems. J. Investig. Derm..

[25] Pinggera A., Mackenroth L., Rump A., Schallner J., Beleggia F., Wollnik B., Striessnig J. (2017). New gain-of-function mutation shows CACNA1D as recurrently mutated gene in autism spectrum disorders and epilepsy. Hum. Mol. Genet..

[26] Scholl U.I., Goh G., Stolting G., de Oliveira R.C., Choi M., Overton J.D., Fonseca A.L., Korah R., Starker L.F., Kunstman J.W. (2013). Somatic and germline CACNA1D calcium channel mutations in aldosterone-producing adenomas and primary aldosteronism. Nat. Genet..

[27] Allely C.S. (2013). Pain sensitivity and observer perception of pain in individuals with autistic spectrum disorder. Sci. World J..

[28] Hoffman T., Bar-Shalita T., Granovsky Y., Gal E., Kalingel-Levi M., Dori Y., Buxbaum C., Yarovinsky N., Weissman-Fogel I. (2022). Indifference or hypersensitivity? Solving the riddle of the pain profile in individuals with autism. PAIN.

[29] Omata K., Anand S.K., Hovelson D.H., Liu C.J., Yamazaki Y., Nakamura Y., Ito S., Satoh F., Sasano H., Rainey W.E. (2017). Aldosterone-Producing Cell Clusters Frequently Harbor Somatic Mutations and Accumulate with Age in Normal Adrenals. J. Endocr. Soc..

[30] Waterhouse A., Bertoni M., Bienert S., Studer G., Tauriello G., Gumienny R., Heer F.T., de Beer T.A.P., Rempfer C., Bordoli L. (2018). SWISS-MODEL: Homology modelling of protein structures and complexes. Nucleic Acids Res..

[31] Seidel E., Schewe J., Scholl U.I. (2019). Genetic causes of primary aldosteronism. Exp. Mol. Med..

[32] Tripolszki K., Gampawar P., Schmidt H., Nagy Z.F., Nagy D., Klivenyi P., Engelhardt J.I., Szell M. (2019). Comprehensive Genetic Analysis of a Hungarian Amyotrophic Lateral Sclerosis Cohort. Front. Genet..

[33] Espino C.M., Lewis C.M., Ortiz S., Dalal M.S., Garlapalli S., Wells K.M., O’Neil D.A., Wilkinson K.A., Griffith T.N. (2022). Na_V_1.1 is essential for proprioceptive signaling and motor behaviors. Elife.

[34] Than K., Kim E., Navarro C., Chu S., Klier N., Occiano A., Ortiz S., Salazar A., Valdespino S.R., Villegas N.K. (2021). Vesicle-released glutamate is necessary to maintain muscle spindle afferent excitability but not dynamic sensitivity in adult mice. J. Physiol..

[35] Carranza Rojo D., Hamiwka L., McMahon J.M., Dibbens L.M., Arsov T., Suls A., Stodberg T., Kelley K., Wirrell E., Appleton B. (2011). De novo SCN1A mutations in migrating partial seizures of infancy. Neurology.

[36] Mantegazza M., Gambardella A., Rusconi R., Schiavon E., Annesi F., Cassulini R.R., Labate A., Carrideo S., Chifari R., Canevini M.P. (2005). Identification of an Nav1.1 sodium channel (SCN1A) loss-of-function mutation associated with familial simple febrile seizures. Proc. Natl. Acad. Sci. USA.

[37] Richards S., Aziz N., Bale S., Bick D., Das S., Gastier-Foster J., Grody W.W., Hegde M., Lyon E., Spector E. (2015). Standards and guidelines for the interpretation of sequence variants: A joint consensus recommendation of the American College of Medical Genetics and Genomics and the Association for Molecular Pathology. Genet. Med..

[38] Kang K.W., Kim W., Cho Y.W., Lee S.K., Jung K.Y., Shin W., Kim D.W., Kim W.J., Lee H.W., Kim W. (2019). Genetic characteristics of non-familial epilepsy. PeerJ.

[39] Rhoads A.R., Friedberg F. (1997). Sequence motifs for calmodulin recognition. FASEB J..

[40] Chan C.S., Guzman J.N., Ilijic E., Mercer J.N., Rick C., Tkatch T., Meredith G.E., Surmeier D.J. (2007). ‘Rejuvenation’ protects neurons in mouse models of Parkinson’s disease. Nature.

[41] Pinggera A., Striessnig J. (2016). Ca(v) 1.3 (CACNA1D) L-type Ca(2+) channel dysfunction in CNS disorders. J. Physiol..

[42] Wang J., Hamill O.P. (2021). Piezo2-peripheral baroreceptor channel expressed in select neurons of the mouse brain: A putative mechanism for synchronizing neural networks by transducing intracranial pressure pulses. J. Integr. Neurosci..

[43] Venugopal S., Ghulam-Jhelani Z., Ahn I.S., Yang X., Wiedau M., Simmons D., Chandler S.H. (2023). Early deficits in GABA inhibition parallels an increase in L-type Ca(2+) currents in the jaw motor neurons of SOD1(G93A) mouse model for ALS. Neurobiol. Dis..

[44] Liss B., Striessnig J. (2019). The Potential of L-Type Calcium Channels as a Drug Target for Neuroprotective Therapy in Parkinson’s Disease. Annu. Rev. Pharm. Toxicol..

[45] Kang S., Cooper G., Dunne S.F., Dusel B., Luan C.H., Surmeier D.J., Silverman R.B. (2012). CaV1.3-selective L-type calcium channel antagonists as potential new therapeutics for Parkinson’s disease. Nat. Commun..

[46] Lamanauskas N., Nistri A. (2008). Riluzole blocks persistent Na+ and Ca2+ currents and modulates release of glutamate via presynaptic NMDA receptors on neonatal rat hypoglossal motoneurons in vitro. Eur. J. Neurosci..

[47] Urbani A., Belluzzi O. (2000). Riluzole inhibits the persistent sodium current in mammalian CNS neurons. Eur. J. Neurosci..

[48] Sonkodi B. (2022). Delayed Onset Muscle Soreness and Critical Neural Microdamage-Derived Neuroinflammation. Biomolecules.

[49] Miller R.G., Mitchell J.D., Moore D.H. (2012). Riluzole for amyotrophic lateral sclerosis (ALS)/motor neuron disease (MND). Cochrane Database Syst. Rev..

[50] Tanaka Y., Yamada M., Koumura A., Sakurai T., Hayashi Y., Kimura A., Hozumi I., Inuzuka T. (2013). Cardiac sympathetic function in the patients with amyotrophic lateral sclerosis: Analysis using cardiac [123I] MIBG scintigraphy. J. Neurol..

[51] Pinto S., Pinto A., De Carvalho M. (2012). Decreased heart rate variability predicts death in amyotrophic lateral sclerosis. Muscle Nerve.

[52] Min S., Chang R.B., Prescott S.L., Beeler B., Joshi N.R., Strochlic D.E., Liberles S.D. (2019). Arterial Baroreceptors Sense Blood Pressure through Decorated Aortic Claws. Cell Rep..

[53] Goldstein D.S., Bentho O., Park M.Y., Sharabi Y. (2011). Low-frequency power of heart rate variability is not a measure of cardiac sympathetic tone but may be a measure of modulation of cardiac autonomic outflows by baroreflexes. Exp. Physiol..

[54] Louradour J., Bortolotti O., Torre E., Bidaud I., Lamb N., Fernandez A., Le Guennec J.Y., Mangoni M.E., Mesirca P. (2022). L-Type Ca(v)1.3 Calcium Channels Are Required for Beta-Adrenergic Triggered Automaticity in Dormant Mouse Sinoatrial Pacemaker Cells. Cells.

[55] Oey P.L., Vos P.E., Wieneke G.H., Wokke J.H., Blankestijn P.J., Karemaker J.M. (2002). Subtle involvement of the sympathetic nervous system in amyotrophic lateral sclerosis. Muscle Nerve.

[56] Sonkodi B., Hegedűs Á., Kopper B., Berkes I. (2022). Significantly Delayed Medium-Latency Response of the Stretch Reflex in Delayed-Onset Muscle Soreness of the Quadriceps Femoris Muscles Is Indicative of Sensory Neuronal Microdamage. J. Funct. Morphol. Kinesiol..

[57] Fang Y., Li Q., Li X., Luo G.H., Kuang S.J., Luo X.S., Li Q.Q., Yang H., Liu Y., Deng C.Y. (2022). Piezo1 Participated in Decreased L-Type Calcium Current Induced by High Hydrostatic Pressure via. CaM/Src/Pitx2 Activation in Atrial Myocytes. Front. Cardiovasc. Med..

[58] Radwani H., Lopez-Gonzalez M.J., Cattaert D., Roca-Lapirot O., Dobremez E., Bouali-Benazzouz R., Eiriksdottir E., Langel U., Favereaux A., Errami M. (2016). Cav1.2 and Cav1.3 L-type calcium channels independently control short- and long-term sensitization to pain. J. Physiol..

[59] Puja G., Sonkodi B., Bardoni R. (2021). Mechanisms of Peripheral and Central Pain Sensitization: Focus on Ocular Pain. Front. Pharm..

[60] Sonkodi B., Bardoni R., Hangody L., Radak Z., Berkes I. (2021). Does Compression Sensory Axonopathy in the Proximal Tibia Contribute to Noncontact Anterior Cruciate Ligament Injury in a Causative Way? A New Theory for the Injury Mechanism. Life.

[61] Rice D.A., McNair P.J., Lewis G.N., Dalbeth N. (2014). Quadriceps arthrogenic muscle inhibition: The effects of experimental knee joint effusion on motor cortex excitability. Arthritis Res..

[62] Comitato A., Bardoni R. (2021). Presynaptic Inhibition of Pain and Touch in the Spinal Cord: From Receptors to Circuits. Int. J. Mol. Sci..

[63] Zhang T.C., Janik J.J., Grill W.M. (2014). Modeling effects of spinal cord stimulation on wide-dynamic range dorsal horn neurons: Influence of stimulation frequency and GABAergic inhibition. J. Neurophysiol..

[64] Foerster B.R., Callaghan B.C., Petrou M., Edden R.A., Chenevert T.L., Feldman E.L. (2012). Decreased motor cortex gamma-aminobutyric acid in amyotrophic lateral sclerosis. Neurology.

[65] Chang Q., Martin L.J. (2011). Glycine receptor channels in spinal motoneurons are abnormal in a transgenic mouse model of amyotrophic lateral sclerosis. J. Neurosci..

[66] Branchereau P., Martin E., Allain A.E., Cazenave W., Supiot L., Hodeib F., Laupenie A., Dalvi U., Zhu H., Cattaert D. (2019). Relaxation of synaptic inhibitory events as a compensatory mechanism in fetal SOD spinal motor networks. Elife.

[67] Torok F., Tezcan K., Filippini L., Fernandez-Quintero M.L., Zanetti L., Liedl K.R., Drexel R.S., Striessnig J., Ortner N.J. (2022). Germline de novo variant F747S extends the phenotypic spectrum of CACNA1D Ca^2+^ channelopathies. Hum. Mol. Genet..

[68] Ding J., Li X., Tian H., Wang L., Guo B., Wang Y., Li W., Wang F., Sun T. (2021). SCN1A Mutation-Beyond Dravet Syndrome: A Systematic Review and Narrative Synthesis. Front. Neurol..

[69] Lossin C. (2009). A catalog of SCN1A variants. Brain Dev..

[70] Huang W., Liu M., Yan S.F., Yan N. (2017). Structure-based assessment of disease-related mutations in human voltage-gated sodium channels. Protein Cell.

[71] Brunklaus A., Brunger T., Feng T., Fons C., Lehikoinen A., Panagiotakaki E., Vintan M.A., Symonds J., Andrew J., Arzimanoglou A. (2022). The gain of function SCN1A disorder spectrum: Novel epilepsy phenotypes and therapeutic implications. Brain.

[72] Carvalho M.D., Swash M. (2009). Awaji diagnostic algorithm increases sensitivity of El Escorial criteria for ALS diagnosis. Amyotroph. Lateral Scler..

[73] Ludolph A., Drory V., Hardiman O., Nakano I., Ravits J., Robberecht W., Shefner J., WFN Research Group on ALS/MND (2015). A revision of the El Escorial criteria—2015. Amyotroph Lateral Scler. Front. Degener..

